# Effect of the Correction of Bilateral Differences in Masseter Muscle Functional Pressure on the Mandible of Growing Rats

**DOI:** 10.3390/jfb14080435

**Published:** 2023-08-21

**Authors:** Shuhei Mizuno, Satoru Matsunaga, Norio Kasahara, Masaaki Kasahara, Yoshiaki Shimoo, Shinichi Abe, Takayoshi Nakano, Takuya Ishimoto, Atsuhiko Hikita, Kunihiko Nojima, Yasushi Nishii

**Affiliations:** 1 Oral Health Science Center, Tokyo Dental College, 2-9-18 Kandamisaki-cho, Chiyoda-ku, Tokyo 101-006, Japan; mizunosyuuhei@tdc.ac.jp (S.M.); nkasahara@tdc.ac.jp (N.K.); kasaharamasaaki@tdc.ac.jp (M.K.); abesh@tdc.ac.jp (S.A.); nojima@ss.iij4u.or.jp (K.N.); nishii@tdc.ac.jp (Y.N.); 2 Department of Orthodontics, Tokyo Dental College, 2-9-18 Kandamisaki-cho, Chiyoda-ku, Tokyo 101-006, Japan; 3 Department of Anatomy, Tokyo Dental College, 2-9-18 Kandamisaki-cho, Chiyoda-ku, Tokyo 101-006, Japan; 4 Department of Histology and Developmental Biology, Tokyo Dental College, 2-9-18 Kandamisaki-cho, Chiyoda-ku, Tokyo 101-006, Japan; 5 Department of Dental Material Science, Tokyo Dental College, 2-9-18 Kandamisaki-cho, Chiyoda-ku, Tokyo 101-006, Japan; 6 Malo Dental and Medical Tokyo, 7-8-10, Chuo-ku, Ginza, Tokyo 104-0061, Japan; implant_zygoma@yahoo.co.jp; 7 Division of Materials & Manufacturing Science, Graduate School of Engineering, Osaka University, 2-1 Yamadaoka, Suita 565-0871, Japan; nakano@mat.eng.osaka-u.ac.jp; 8 Aluminium Research Center, University of Toyama, 3190 Gofuku, Toyama 930-8555, Japan; ishimoto@sus.u-toyama.ac.jp; 9 Department of Tissue Engineering, The University of Tokyo Hospital, Hongo 7-3-1, Bunkyo-ku, Tokyo 113-8655, Japan; ahikita-tky@umin.ac.jp

**Keywords:** bone quality, collagen fibre, biological apatite crystallite, microbeam X-ray diffraction, second harmonic generation imaging

## Abstract

The objective of this study is to clarify the effect of restoring the lowered masticatory muscle functional pressure and correcting bilateral differences in masticatory muscle functional pressure on jawbone growth during growth and development with a quantitative evaluation of the changes in the micro/nanostructural characteristics of entheses. Male Wistar rats aged 4 weeks were divided into an experimental group injected with a botulinum toxin serotype A (BoNT/A) formulation to reduce muscle function (BTX group) and a control group (CTRL group). They were euthanised after 6, 8, 10, 12, and 16 weeks after measuring the difference between the midline of the upper and lower incisors. The mandibles were harvested for histological examination, second harmonic generation imaging, and the quantitative evaluation of biological apatite (BAp) crystal alignment. The midline difference decreased with age in weeks. In rats from 6 weeks after BoNT/A administration to 12 weeks after administration, the collagen fibre bundle diameter was significantly smaller in the BTX group; the difference between the two groups decreased with increasing age. BAp crystal alignment was significantly different on the *x*-axis and the *y*-axis on the BTX group from 6 weeks after BoNT/A administration to 10 weeks after administration. Asymmetry of mandibular bone formation caused by load imbalance during growth could be corrected by the adjustment of the function of the masseter muscle on either side.

## 1. Introduction

The growth and development of the dentition and the jawbone are influenced by genetic and nongenetic environmental factors [1,2]. Enlow et al. reported that the growth and development of the region above the base of the nasal cavity is related to the growth of the cranial base, sutural growth, and the development of respiratory function, whereas the growth and development below the base of the nasal cavity is closely linked to the mandibular function from the masticatory muscles [3,4,5]. In particular, the acquisition of masticatory muscle function is thought to play a major role in the growth and development of the lower face [6,7]. It has been shown that there is a relationship between abnormal function of the masticatory muscles and the underdevelopment of the jawbone during the period of growth and development, suggesting that imbalance in the function of the masticatory muscles on either side may be an environmental factor causing malocclusion [8,9]. Dysfunction of the perioral muscles, as an environmental factor, can adversely affect oral functions, such as chewing, swallowing, pronunciation, and breathing, and can cause an imbalance in perioral muscle functional pressure, which in turn affects dental morphology [10]. Furthermore, it can inhibit normal jawbone growth and development in children, causing oral–facial morphology abnormalities [11]. Oral myofunctional disorders are environmental factors in the development of malocclusion and also cause dental problems, such as retroversion of orthodontic treatment, periodontal disease, and noncompliance with dental prosthetics [12,13]. Stahl et al. reported that children with malocclusion and perioral muscle disharmony are common, with a prevalence of oral–facial dysfunction of 61.6% in children with deciduous dentition and 80.8% in children with mixed dentition [14].

In view of this, the correct adjustment of abnormal muscle function pressure is thought to promote the healthy growth of the jawbone as well as the dentition, and is widely practiced as oral myofunctional therapy (MFT) [15]. Improving the function of the perioral muscles through MFT is one solution to the problems caused by imbalances in oral muscle functional pressure, as it creates an environment that maintains the normal morphology of the jawbone and dentition [11]. MFT involves training individual muscles, chewing, swallowing, pronunciation, and resting lip and tongue position to eliminate laxity and overstrain of the perioral muscles, and to balance and maintain functional pressure throughout the dentition [16]. It is believed that improving oral function leads to the removal of environmental factors that establish malocclusion, and thus the dentition and occlusion are naturally guided to a normal form without the use of orthodontic appliances, and that the combination of orthodontic treatment and MFT can provide stability of the dentition after treatment [12]. However, the mechanical environment regulating the homeostasis of the jawbone is complex, and further evidence based on quantitative evaluations is needed to ensure effective MFT.

There have been numerous attempts to quantitatively evaluate the mechanical environment of bone, and analytical methods have been developed for bone mass and other bone morphometry [17,18]. In particular, since it was reported that the micro/nanostructural properties of bone are greatly reflected in bone strength, research using material engineering methods for the analysis of bone quality has been carried out to accurately predict the mechanical function of bone [19,20,21]. Studies have shown that the main extracellular matrix of collagen fibres and biological apatite (BAp) crystals is a major factor in the resistance to stress on bony tissue, and that collagen fibres resist tensile stress, whereas BAp crystals resist compressive stress [22,23]. Warshaw et al. showed that the mechanical environment and anisotropy of collagen fibre orientations within the bone are important factors reflecting the mechanical function of bone in the direction of the load [24]. On the other hand, Nakano et al. used microbeam X-ray diffraction analysis to quantitatively evaluate BAp crystal orientation in whole-body bone in experimental animals and found a high correlation between bone strength and BAp crystal orientation [17,18]. Since bone quality factors strongly reflect the local loading environment, it is possible to predict the mechanical function of the jawbone with a high degree of accuracy by the quantitative evaluation of the histomorphology and anisotropy of the micro/nanostructure of entheses of the masticatory muscles attachment.

Seok et al. reported that the botulinum-toxin-induced imbalance of bilateral masticatory muscle functional pressure affects mandibular growth and causes jaw deformity in growing rats [25]. Kusaba et al. studied a model of unilateral masticatory muscle hypofunction induced by botulinum toxin, and they found histological changes and decreased bone quality at the entheses where the functional pressure had been reduced. They suggest that this structural change may be involved in the deformation of the jawbone during growth and development [26].

The objective of this study was therefore to perform a quantitative evaluation of the changes in the micro/nanostructural characteristics of entheses in growing rats with reduced unilateral masticatory muscle functional pressure that was subsequently restored, with the aim of clarifying what effect the correction of bilateral differences in masticatory muscle functional pressure that occurred during the early growth period may have on the deformation of the jaw that has already developed.

## 2. Materials and Methods

This animal experiment was conducted in accordance with the guidelines for animal experiments of Tokyo Dental College (ethical review application no. 213105). Invasive procedures were kept to a minimum to reduce suffering in the experimental animals.

### 2.1. Experimental Animals

A total of 100, 4-week-old, male, Wistar rats were divided into an experimental group (BTX group, *n* = 50), which were injected with Botulinum toxin serotype A (BoNT/A; Botox^®^, Allergan Pharmaceuticals, Dublin, Ireland), and a control group (CTRL group, *n* = 50). The experimental animals were reared in plastic cages with free access to water and solid food. Rats with significantly lower than standard body weight, scarring, pathological alopecia, or malocclusion were excluded from the experiment.

### 2.2. Experimental Procedures

**Figure 1:**
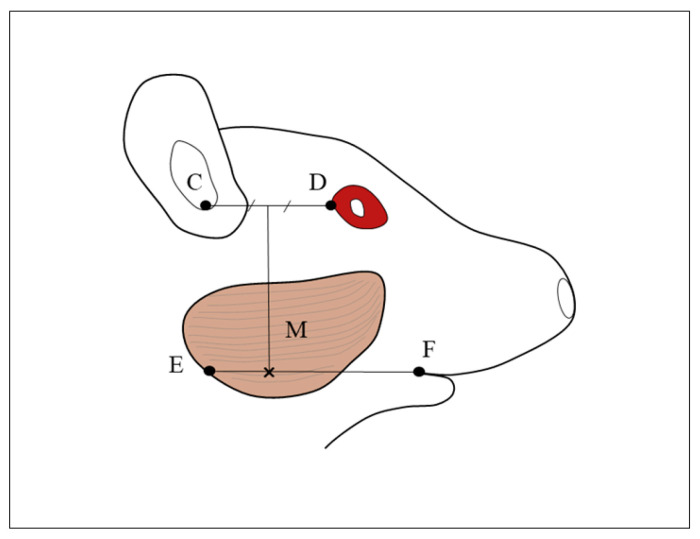
Cross marks indicate the injection site. M: masseter muscle; C: auditory meatus; D: lateral canthus; E: Most posterior point of mandibular angle; F: outer oral commissure. The injection site in the masseter muscle is the point where the line perpendicular from the line CD at its midpoint intersects with the line EF “Reprinted/adapted with permission from Ref. [26]. 2021, The Hard Tissue Biology Network Association (JHTBNet)”.

The experimental animals were given general anaesthesia by intraperitoneal administration of a combination of three anaesthetics (medetomidine hydrochloride, 0.75 mg/kg, Nippon Zenyaku Kogyo Co., Ltd., Fukushima, Japan; midazolam, 4.0 mg/kg, Sandoz K.K., Tokyo, Japan; butorphanol tartrate, 5.0 mg/kg, Meiji Seika Pharma Co., Ltd., Tokyo, Japan). Rats in the BTX group were injected in the right masseter at the angle of the mandible with 5.0 U (0.3 mL) of BoNT/A diluted with physiological saline (Figure 1). Rats in the CTRL group were injected in the right masseter with 0.3 mL of physiological saline. In all rats, no difference in the upper and lower incisor midline was confirmed before injection. The rats were reared for 6, 8, 10, 12, or 16 weeks and then euthanised under deep anaesthesia, and the mandibles were harvested for examination. Before euthanasia, the sagittal suture and the upper incisor midline were checked to ensure that they were in the same plane, and the difference between the upper and lower incisor midlines was measured with callipers in occlusion.

### 2.3. Measurement of Weight

Following euthanasia, the rats were weighed, the masseter was harvested from the origin of the muscle on the zygomatic arch and the insertion on the mandible, and the muscle was weighed.

### 2.4. Setting the Region of Interest

**Figure 2:**
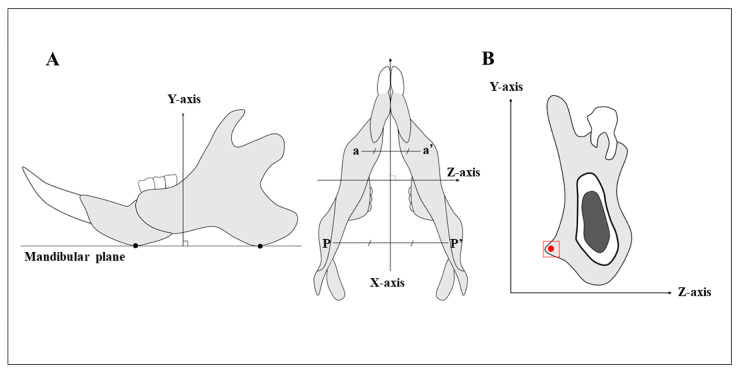
(**A**) Point a is the lowest point in the anterior thick area of the mandible, and point p is the lowest point in the posterior thick area. The mandibular plane passes through the lines a-a′ and p-p′. The *x*-axis passes through the midpoints of the lines a-a′ and p-p′. The *y*-axis is the axis perpendicular to the mandibular plane, and the *z*-axis is the axis perpendicular to *x*-*y*. (**B**) The *YZ* plane of a mandibular third molar passing through the centre of the crown. The red box shows the region of histological observation. The red dot shows the measurement point of BAp crystal alignment. The measurement point is the attachment site of the masseter muscle in the area of the mandibular third molar “Reprinted/adapted with permission from Ref. [26]. 2021, The Hard Tissue Biology Network Association (JHTBNet)”.

The basic axes of the mandibles were set by taking the mesiodistal direction as the *x*-axis, the direction perpendicular to the mandibular plane as the *y*-axis, and the labiolingual direction as the *z*-axis (Figure 2A). The region of interest was set as the mandibular third molar region (Figure 2B).

### 2.5. Tissue Slice Preparation

The BTX group (*n* = 50) and control group (*n* = 50) were divided into the paraffin-block-making group (*n* = 25) and the masseter-muscle-harvesting and resin-block-making group (*n* = 25).

#### 2.5.1. Paraffin Section

The specimens were fixed in 4% paraformaldehyde phosphate buffer solution and decalcified for 4 weeks in 10% ethylenediaminetetraacetic acid. They were then embedded in paraffin according to the usual method. Thin slices were taken along the *YZ* plane (frontal section), and they were then stained by Masson’s trichrome method. After staining, the thickness of the tendon at the tendon–bone margin was measured (*n* = 5).

#### 2.5.2. Masseter Muscle Section and Resin Section

The right masseter muscle was harvested and stained with haematoxylin and eosin, and the mean cross-sectional area of each masseter muscle fibre in an area of 500 μm^2^ centred on the injection site was calculated (*n* = 5). Specimens were also embedded in cold-curing acrylic resin (SCANDIPLEX, SCAN-DIA, Hagen, Germany) for the preparation of polished specimens. The cured blocks were sliced in the *XY* plane through the 3rd molar region using a saw microtome (SP1600, Leica, Wetzlar, Germany) with a blade width of 300 µm. The slices were then sanded using waterproof abrasive paper (#400, #800, and #1200) to produce polished specimens with 200 µm in thickness.

### 2.6. Second Harmonic Generation Imaging

Second harmonic generation (SHG) images were acquired using a multiphoton confocal microscope system (LSM880 NLO, Carl Zeiss, Jena, Germany) with an excitation laser (Chameleon Vision II; wavelengths: 680–1080 nm, repetition rate: 80 MHz, pulse width: 140 fs; Coherent Inc., Santa Clara, CA, USA) and an objective lens (Plan-Apochromat 20×/0.8 M27, Carl Zeiss). Following image acquisition, the collagen fibre bundles were quantitatively evaluated using Imaris 8.4 software (Bitplane AG, Zurich, Switzerland). Collagen fibres with a thickness greater than 375 nm, which can be imaged with multiphoton excitation phase-contrast microscopy, were extracted as collagen fibre bundles. Evaluation was carried out by tracing the collagen fibre bundles within the bone in approximately 200 µm^2^ of the mandibular masseter muscle prominence and calculating the mean diameter [27].

### 2.7. BAp Crystal Alignment

BAp crystal alignment was quantitatively evaluated using an optical curved imaging plate (IP) X-ray diffractometer (XRD; D/MAX RAPID II-CMF, Rigaku Corporation, Tokyo, Japan). Measurements were taken from the masseter muscle attachment at the third molar of 200 µm thick, undecalcified, polished specimens. X-rays were irradiated using an optical microscope (×0.6–4.8 magnification) attached to the XRD so that the incident beam was circular with a diameter of 100 µm. Measurements were made in the *x*-axis direction using the reflecting optical system, and in the *y*- and *z*-axis directions using the transmission optical system, both using Cu-Kα radiation as the beam source. The tube voltage was set to 40 kV, with the tube current at 30 mA, and the diffracted X-ray beam was detected using a curved IP. The analysis conditions were set according to the method of Nakano et al. [18]. The 2D Data Processing Software ver. 2.1.6 (Rigaku, Tokyo, Japan) was used to calculate the X-ray intensity ratios of the two diffraction peaks in the 002 and 310 planes from the diffraction ring images.

### 2.8. Statistical Analysis

For statistical analysis, means were calculated, and the one-way analysis of variance (ANOVA) and *t*-test were used. A *p* < 0.05 was considered statistically significant.

## 3. Results

### 3.1. Incisor Midline

**Figure 3:**
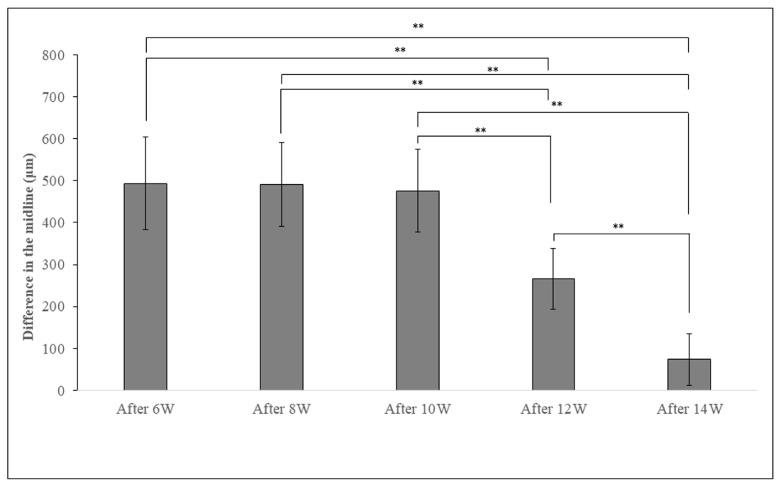
Measurement of the difference between the upper and lower incisor midline in the BTX group. As the weeks passed, the midline difference between the upper and lower incisors became smaller. The data are means ± standard deviation in independent experiments with *n* = 5. Comparison with the CTRL group, ** *p* < 0.01.

In the BTX group, there was a large difference in the midline of the upper and lower incisors. The difference in the midline decreased with each passing week and was significantly smaller after 12 weeks of BoNT/A administration compared to 6, 8, and 10 weeks after Botox administration (Figure 3).

### 3.2. Body Weight

**Figure 4:**
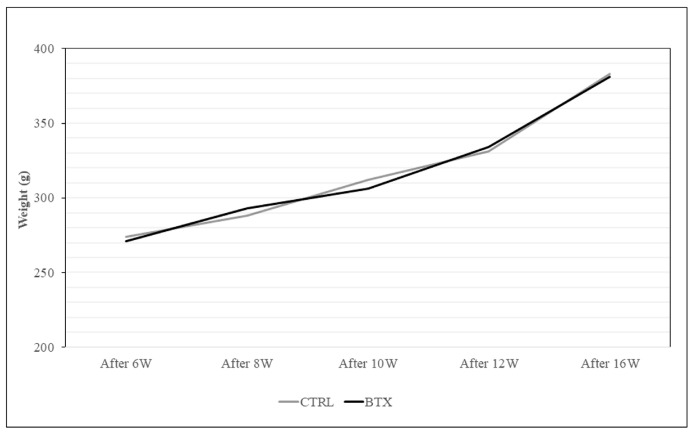
Body weight measurement. Both BTX and control groups increased their body weight as the weeks passed. The data are means ± standard deviation in independent experiments with *n* = 10.

Body weight increased with the age of the rats. No significant difference in body weight was found between the BTX group and the CTRL group (Figure 4).

### 3.3. Masseter Muscle Weight

**Figure 5:**
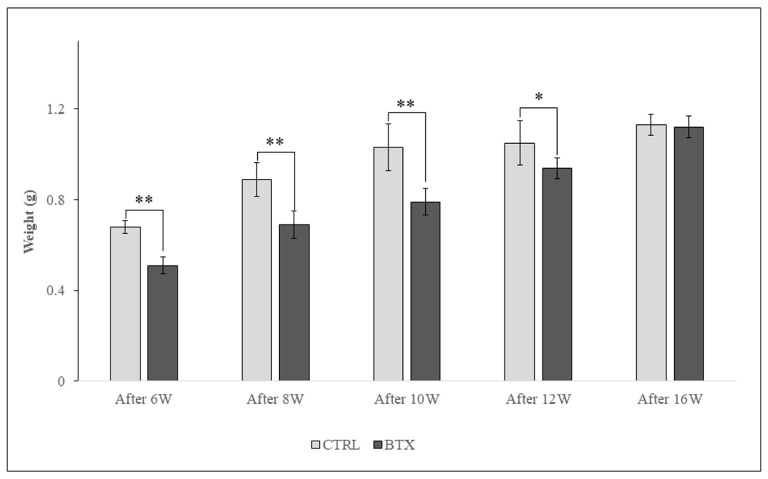
Masseter muscle weight measurement. Significantly lower in the BTX group compared to the CTRL group. The difference between the two groups decreased as the weeks of age progressed. The data are means ± standard deviation in independent experiments with *n* = 5. Comparison with the CTRL group, * *p* < 0.05, ** *p* < 0.01.

Masseter muscle weight increased with the age of the rats. From 6 weeks after BoNT/A administration to 12 weeks after administration, masseter muscle weight was significantly lower in the BTX group than in the CTRL group. At 16 weeks after administration, there was no significant difference in masseter muscle weight between the two groups (Figure 5).

### 3.4. Histological Observation of Masseter Muscle Fibres

**Figure 6:**
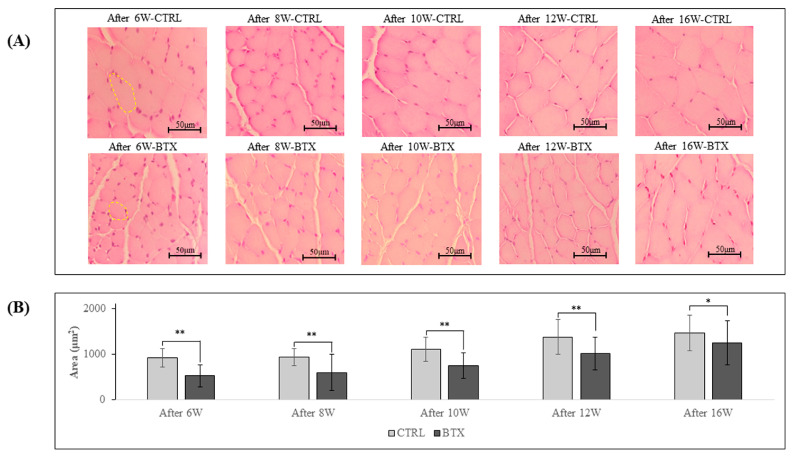
(**A**) Haematoxylin–eosin-stained masseter muscle fibres. Yellow border: Masseter muscle fibres. (**B**) Cross-sectional area of masseter muscle fibres. Masseter muscle fibres in the BTX group show atrophy. The data are means ± standard deviation in independent experiments with *n* = 5. Comparison with the CTRL group, * *p* < 0.05, ** *p* < 0.01. Scale bar: 50 μm.

In the CTRL group, the masseter muscle fibres were closely packed with very little space between them, whereas in the BTX group, there were wide gaps between the muscle fibres and the masseter muscle fibres showed atrophy (Figure 6A). At each age, the cross-sectional area of the masseter muscle was significantly smaller in the BTX group than in the CTRL group (Figure 6B).

### 3.5. Histological Observations at the Entheses

**Figure 7:**
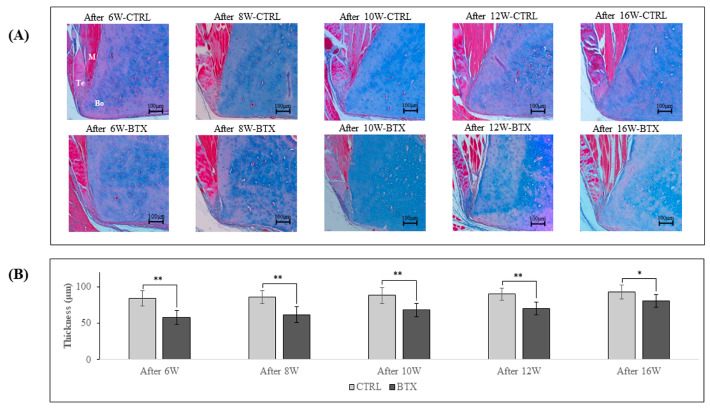
(**A**) Masson’s trichrome-stained *YZ* cross-sections of rat mandibles in the third molar region. M: masseter; Bo: bone (mandible); Te: tendon. The masseter muscle is attached to the mandible by a tendinous insertion at the inferior margin of the masseter muscle prominence and adheres to the mandible via the periosteum at all other sites. (**B**) Thickness of the tendon. The data are means ± standard deviation in independent experiments with *n* = 5. Comparison with the CTRL group, * *p* < 0.05, ** *p* < 0.01. Scale bar: 100 μm.

Figure 7 shows images of Masson’s trichrome staining of the third molar region of frontal sections of 6, 8, 10, 12, and 16 weeks after BoNT/A administration rats. At all ages, the masseter muscle was attached to the mandible by a tendinous insertion at the inferior margin of the masseter muscle prominence and adhered to the mandible via the periosteum at all other sites. The thickness of the ligament was significantly lower in the BTX group than in the group at all ages.

### 3.6. Anisotropy of Collagen Fibre Orientation

**Figure 8:**
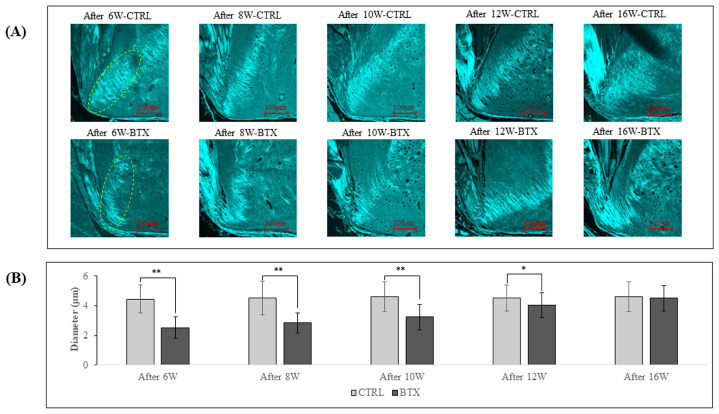
(**A**) SHG images of *YZ* cross-sections of rat mandibles at the third molar region. The green fluorescent areas are collagen fibre bundles. (**B**) Diameters of collagen fibre bundles. The collagen fibre bundles show a significantly smaller diameter in the BTX group from 6 weeks after BoNT/A administration to 12 weeks after administration. The data are means ± standard deviation in independent experiments with *n* = 5. Comparison with the CTRL group, * *p* < 0.05, ** *p* < 0.01. Scale bar: 100 μm.

Figure 8 shows SHG imaging of the third molar region. In rats from 6 weeks after BoNT/A administration to 12 weeks after administration, the diameter of the collagen fibre bundles was significantly smaller in the BTX group than in the CTRL group. The difference between the two groups grew less with increasing age, and there was no significant difference between the groups at 16 weeks after administration.

### 3.7. BAp Crystal Alignment

**Figure 9:**
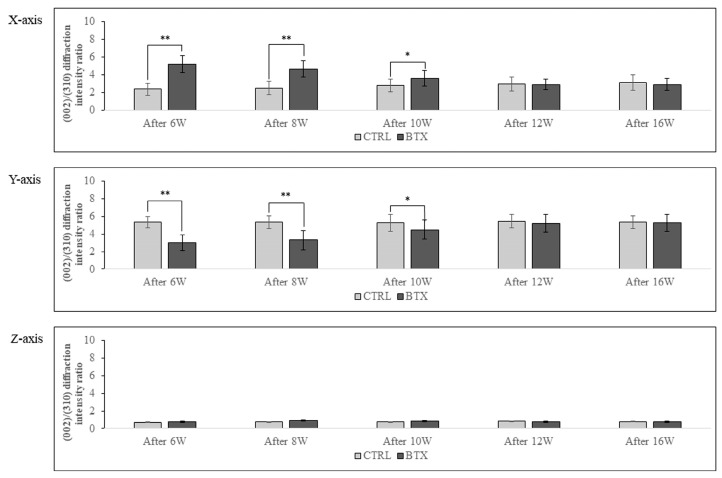
BAp crystal alignment in *x*-, *y*- and *z*-axis. different between the *x*-axis and the *y*-axis in the BTX group from 6 weeks after BoNT/A administration to 10 weeks after administration. The data are means ± standard deviation in independent experiments with *n* = 5. Comparison with the CTRL group, * *p* < 0.05, ** *p* < 0.01.

Figure 9 shows the calculated X-ray diffraction intensity ratio on the *x*-, *y*-, and *z*-axes at the entheses. The X-ray diffraction intensity ratio of hydroxyapatite powder was 1.04 with the reflecting system and 3.13 with the transmission system. On the *x*-axis, the X-ray diffraction intensity ratio was significantly higher in the BTX group than in the CTRL group in rats 6, 8, and 10 weeks after BoNT/A administration. On the *y*-axis, the X-ray diffraction intensity ratio was significantly lower in the BTX group than in the CTRL group in rats 6, 8, and 10 weeks after administration. However, there were no significant differences between the BTX and CTRL groups on either the *x*- or the *y*-axis at 12 or 16 weeks after administration. On the *z*-axis, both groups showed extremely low values for the X-ray diffraction intensity ratio.

## 4. Discussion

The jawbone and dentition are constantly surrounded by muscles and are always under pressure from the muscles. Since the forces of the tongue and lips and cheeks are applied to the dentition from the inside and outside, respectively, the pressures from these perioral muscles must be balanced in order to maintain a healthy dentition and bite over the long term [10]. Dysfunction of the perioral muscles causes problems in basic functions of the oral cavity, such as chewing, swallowing, and pronunciation. In addition, perioral muscle dysfunction inhibits the normal development of the jawbone and dentition, and is considered a cause of malocclusion [14]. In orthodontic treatment, it is believed that MFT and functional orthodontic appliances can be used to remove dysfunctions of the perioral muscles [15]. Individual muscle training includes training for each region, such as the tongue, lips, facial expression muscle group, and masticatory muscle group, and it is believed that training appropriate to the condition of each muscle can create an environment to maintain normal morphology of the jawbone and dentition [12]. Malta et al. suggest that orthodontic treatment with functional orthodontic appliances maintains good long-term results due to a combination of skeletal, dental, and soft tissue changes [28]. Tulloch et al. suggest that early orthodontic treatment is effective when approaching skeletal imbalances [29].

BoNT/A preparations are composed primarily of bacterial metalloproteinases. This proteolytic enzyme can specifically inhibit neurotransmitter release in cholinergic nerve endings, causing prolonged muscle relaxation [30]. Tsai et al. used electromyography signals to follow the changes in masseter muscle activity following the administration of BoNT/A in adult rats, and they reported a tendency for muscle function to recover [31]. In the present study, masseter muscle weight was lower in the BTX group than in the CTRL group until 12 weeks after BoNT/A administration, but there was no significant difference between the groups at 16 weeks after administration. The masseter muscle cross-sectional area was smaller in the BTX group than in the CTRL group at all ages, but the difference between the groups decreased with increasing age. The measurement of the upper and lower incisor medians showed a large discrepancy between the upper and lower medians in the BTX group, but the difference in the median line decreased with each passing week. Tsai et al. suggested that the decrease in masseter muscle weight associated with the administration of BoNT/A reduced muscle pressure, and that this load reduction led to jawbone deformity [31,32,33], and the results of this suggested that the changes in the midline of the upper and lower incisors in the study originated from changes in the functional pressure of the masseter muscle.

Next, the bone was analysed by examining collagen fibre bundles, which exhibit resistance to tensile stress. As reported by Chong et al. [34], band-like collagen fibre bundles were formed in the bone with growth, and in the CTRL group, the intrabone collagen fibre bundles in the masseter muscle attachment area were thick and almost coincided with the direction of running tendon fibres. At the same time, Kusaba et al. reported that, if there is a persistent reduction in functional pressure of the masseter muscle during the period of growth and development, collagen fibre bundles are not generated in sufficient quantity or quality within the entheses [26]. In the present study, the BTX group showed less thickness of the collagen fibre bundles than the CTRL group until 12 weeks after BoNT/A administration, and at 16 weeks after administration, there was no significant difference between the two groups in the thickness of the collagen fibre bundles. These results suggest that abundant collagen fibre bundles were again generated at the entheses as a result of the restoration of masseter muscle function. The preferential alignment of BAp crystals, which exhibit resistance to compressive stress, was also examined. BAp crystals are ionic crystals with a highly anisotropic hexagonal nanostructure, and the c-axis of the crystal is preferentially aligned with the direction of loading [35]. BAp crystals in the mandible basically show preferential, single-axis alignment along the long axis, but near the root of the tooth, the orientation in the long axis direction decreases and the crystals show preferential alignment in the direction of mastication [17]. This suggests that BAp crystals acquire preferential alignment in cortical bone reflecting the in vivo stress distribution at the various sites within the bone. Bacon et al. reported that BAp crystals at the entheses are preferentially aligned with the running direction of the muscle [36]. Kusaba et al. found that the orientation of BAp crystals was less closely aligned with the running direction of the muscle and tendon in the mandibles of growing rats that had been treated with Botox [26]. In the present study as well, BAp crystal alignment with the running direction of the muscle and tendon in rat mandibles was reduced until 10 weeks after BoNT/A administration, but at 12 weeks after administration, the BAp crystals showed preferential alignment with the running direction of the muscle and tendon. These results suggest that the BAp crystal alignment that should normally have been acquired was obtained as a result of recovery of masseter muscle functional pressure.

The work of Tsai et al. suggests that a decrease in masseter muscle weight is associated with a reduction in muscle functional pressure, and this reduction in load leads to the deformation of the jawbone [31,32,33], which is grounds for the notion that imbalance in muscle functional pressure is a factor in jaw deformity.

## 5. Conclusions

The results of this study indicate that botulinum-toxin-induced differences in the midline of the upper and lower incisors can be improved in growing rats. It was suggested that the loss of masticatory muscle functional pressure causes jaw deformity, and that jaw deformity returns when functional pressure is restored.

## Data Availability

Raw data were generated at Oral Health Science Center in Tokyo Dental College. Derived data supporting the findings of this study are available from the corresponding author [S. Matsunaga] on request.
